# Epidemiological Characteristics of the COVID-19 Pandemic During the First and Second Waves in Chhattisgarh, Central India: A Comparative Analysis

**DOI:** 10.7759/cureus.24131

**Published:** 2022-04-13

**Authors:** Pragya Agarwala, Anudita Bhargava, Dharmendra Kumar Gahwai, Sanjay Singh Negi, Priyanka Shukla, Sonal Dayama

**Affiliations:** 1 Microbiology, All India Institute of Medical Sciences, Raipur, Raipur, IND; 2 Directorate of Health Services, Government of Chhattisgarh, Raipur, IND; 3 Directorate of Health & Family Welfare, Government of Chhattisgarh, Raipur, IND

**Keywords:** surveillance, waves, epidemiology, comparative analysis, covid-19

## Abstract

Background

There is a scarcity of reports of comparative analysis between the first and second waves of the pandemic from any part of India. This article aims to comprehensively investigate the epidemiology of coronavirus disease 2019 (COVID-19) during the course of the pandemic in the state of Chhattisgarh, central India.

Methodology

A comparative epidemiological analysis of the first and second waves of COVID-19 across Chhattisgarh was conducted on the vital parameters of total tests performed, cases diagnosed, age and gender distribution, case fatality ratio (CFR), and mitigation strategy reported by the state and central government health agencies using the data from Indian Council of Medical Research and National Informatics Centre portals.

Results

The second wave was shorter than the first wave but the absolute number of cases increased by 2.4 times and deaths by 2.7 times. There was a significant increase in cases per million, deaths per million, and test positivity rate. The hospitalization rate and test per case ratio dropped in the second wave from 33 to 20 and from 12.6 to 7.2, respectively. Both infection and deaths were higher among males in both the waves (p < 0.001). CFR increased from 1.2% in the first wave to 1.4% in the second wave (p < 0.001; odds ratio = 1.14 (1.1-1.19)). Increased mortality was seen in all ages except the young (≤20 years) and the old (>60 years).

Conclusions

The significantly high number of cases and deaths during the second wave provides evidence to undertake preparedness measures for mitigating any future waves. Regular surveillance, monitoring, and analysis of epidemiological data are pertinent for continued situational awareness.

## Introduction

More than one year after the declaration of the pandemic, coronavirus disease 2019 (COVID-19), caused by the severe acute respiratory syndrome coronavirus 2 (SARS-CoV-2), continues to wreak havoc across the globe. With mere 536 reported cases and 11 deaths on March 24, 2020, India promptly announced a strict nationwide lockdown extended in phases, bringing about the much-desired flattening of the curve initially. Economic challenges led to a staggered unlock strategy, progressively increasing social interaction and commercial activity, with the subsequent months witnessing a massive upsurge of cases with an exponential growth rate. The peak of the first wave of the pandemic was in mid-September when there were more than 10 lakh active cases in the country, with the highest single-day spike of 97,894 new cases recorded on September 16, 2020 [1]. Thereafter, there was a steady decline in the number of new cases per day during the next couple of months. On February 16, 2021, the country reported a mere 9,000 new cases and an active caseload of 1.36 lakhs [2]. The vaccination program in India began on January 16, 2021, and it was widely believed that the battle against the virus was soon ending. By the end of February 2021, there was a sharp rise in the number of daily reported cases, which culminated in the second wave of the pandemic. Relaxation of interventions, negligence in public behavior, waning immunity, some superspreader religious and political events, and the emergence of more transmissible variants (B.1.617 lineage) are the various reasons that led to the onset of the second wave [3]. The second wave of the pandemic in India brought about a tsunami of cases, with health systems getting overwhelmed in most parts of the country [3]. The second wave of the pandemic also displayed wide disparities in geographical distribution, with a few states peaking much ahead of others [4].

Chhattisgarh, a state located in central India, has a population of 30 million spread across 28 administrative units called districts. The state of Chhattisgarh reported its first case of COVID-19 on March 19, 2020, in an international traveler. By the time the nationwide lockdown was instituted on March 25, 2020, Chhattisgarh had three cases. In the next three months (March 19 to June 30, 2020), the number of cases slowly but steadily increased to 2,911. Subsequently, Chhattisgarh witnessed its peak of cases in September and October 2020. Thereafter, the number of cases decreased steadily. However, Chhattisgarh was one of the first Indian states to show a steep increase in cases and test positivity rates during the early phase of the second wave [4]. The number of new cases reported per day in Chhattisgarh sharply escalated from 475 on March 15, 2021, to 3,108 on March 31 to 14,098 on April 11, 2021 [5]. The second wave of COVID-19 ended around June in most parts of the country. Thereafter, cases decreased steadily and vaccination moved at a fast pace, and it was speculated that the pandemic was largely over unless some immunity-escaping mutants arise. Cases in India started increasing by the end of December 2021 with the emergence of the Omicron variant of the virus. The third wave of the pandemic waned by March 2022 in India and Chhattisgarh.

In this study, we describe the comparative epidemiologic features of the first and second waves of the COVID-19 pandemic in the state of Chhattisgarh. This holds importance as it is probably one among few states to have analyzed and presented the humongous data of COVID-19 scientifically.

## Materials and methods

Statewide COVID-19 data from March 06, 2020, to June 30, 2021, were collected from the State Surveillance Unit (SSU), Integrated Disease Surveillance Programme (IDSP), Chhattisgarh, which included daily incidence of laboratory-confirmed cases, number of fatalities, and patient demographics. SSU guides, supervises, and monitors the activities of District Surveillance Units. Since the onset of the pandemic, the Indian Council of Medical Research (ICMR) and the National Centre for Disease Control, under the leadership of the Ministry of Health and Family Welfare (MoHFW), the Government of India, is guiding the country on COVID-19 testing and surveillance activities. The data collected at the time of obtaining samples and testing is entered onto a centralized ICMR portal. This is in the form of a standard Sample Registration Form developed by ICMR and is uniform across the entire country and is available on its website (www.icmr.gov.in). Samples were collected from various high-risk groups (such as travelers at the airport, railway station, bus transit points); routine symptomatic screening in the community through frontline health workers; surveillance of all contacts of a positive case; screening of vendors; COVID-19 warriors such as sanitary workers, police personnel, bankers, health professionals; and those who came to health facilities for COVID testing. After a case was registered positive on the ICMR portal, it was imported onto a portal developed by the Chhattisgarh Health Department in collaboration with the National Informatics Centre for managing data on hospital admissions, home isolation, and mortality of COVID-19-positive cases. These data were used by administrators and policymakers for dissemination to the general community daily in bulletin form and for management purposes. For this study, data downloaded from these portals were used (www.icmr.gov.in, www.cghealth.nic.in). The start point of each wave for Chhattisgarh was set as the first date when two consecutive seven-day rolling averages of new COVID-19 cases were >500, and the endpoint was defined as the last day when the number fell below 500 cases for two consecutive days. Rolling averages were used because they eliminate periodic fluctuations and provide a more consistent series than daily observations.

Descriptive analysis was performed using MS Office Excel and Epi Info. Metrics such as test positivity rate (TPR) and case fatality ratio (CFR) were compared with Indian statistics available on MoHFW and ICMR websites. Demographics and CFR were compared between the first and second waves. The chi-square test and standard error of proportions were used for categorical variables, male and female deaths, and a p-value of <0.001 was considered statistically significant. The following standard formulae were used for the analyses: (a) CFR (%) = Number of deaths/Number of cases × 100. (b) TPR (%) = Number of positive (COVID-19) tests/Number of samples tested × 100. (c) Cases per million population = Total number of confirmed COVID-19 cases/Total population × 1,000,000. (d) Tests per million population = Total number of tests conducted/Total population × 1,000,000. (e) Deaths per million population = Total number of COVID-19 deaths/Total population × 1,000,000. (f) Age-specific mortality = Number of deaths in a particular age group due to COVID-19/Number of positive cases in that age group. For example, age-specific mortality in the age group of 60-70 years = Number of COVID-19 deaths in the age group of 60-70 years/Number of positive cases in the age group of 60-70 years.

This study was exempted from review by the Institutional Ethical Committee, All India Institute of Medical Sciences, Raipur (1943/IEC-AIIMSRPR/2021) as it analyzed data already available in the public domain.

## Results

**Figure 1:**
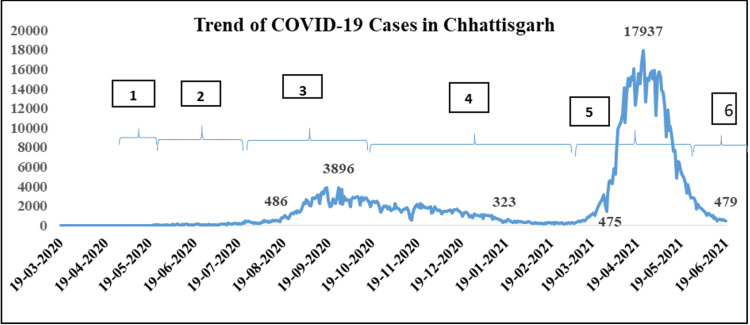
Trend of COVID-19 cases in the state of Chhattisgarh along with timelines of containment measures. 1. March 25 to May 17, 2020: Nationwide lockdown, strict restrictions. 2. May 18 to July 21, 2020: National and state restrictions eased. 3. July 22 to September 28, 2020: Complete lockdown in major cities and partial restrictions in smaller districts. 4. September 28 to April 12, 2021: Partial restrictions remain. Schools were still closed. 5. April 13 to May 31, 2021: Lockdown imposed in districts with a TPR of more than 8. 6. June 01, 2021, onwards: Restrictions eased, except in districts with a TPR of >5. COVID-19: coronavirus disease 2019; TPR: test positivity rate

After the first case in Chhattisgarh was reported on March 19, 2020, in an international traveler [6], the nationwide phasic lockdown implemented from March 25 to May 17, 2020, kept the number of COVID-19 cases in a tight check in Chhattisgarh. The two consecutive months of unlocking brought about a slow but steady rise in the number of new cases per day. The average daily cases increased from 200 in the first week of July to 700 in August, 2,000 in September, and 2,500 in October. The first wave of the pandemic started on August 15, 2020, and continued till January 17, 2021, lasting 156 days (Figure 1). The wave reached its peak on September 26 with 3,896 new cases identified and declined slowly with periodic rises and dips (Figure 1). The month of February 2021 witnessed a consistent average daily new cases of fewer than 250, in tune with the scenario in most parts of the country. The second wave of the COVID-19 pandemic began on March 13, 2021, peaked on April 23 (17,937 cases), and ended on June 19, 2021, lasting 99 days.

Cases and deaths

The daily cases in April 2021 were more than four times the peak numbers in 2020. Similarly, the number of fatalities also increased up to 3.8 times. In September 2020, which was the peak month during the first wave, there was an average of 39 deaths daily, whereas, in April 2021, the peak month of the second wave, the average daily death toll rose to 147. The CFR increased from 0.2% in May 2020 to 0.8% in September 2020 and later remained consistent at 1.2% from November 2020 to April 2021. In May and June 2021, it increased to 1.4%. In 2020, the monthly CFR was the highest in August (1.7%) and September (1.4%), and in 2021, it was the highest in January (1.4%), February (1.8%), May (1.8%), and June (1.7%) (Appendices).

Tests and positivity rate

Chhattisgarh had no laboratory to conduct reverse transcriptase-polymerase chain reaction (RT-PCR) tests on COVID-19 suspects at the outset of the pandemic. Test samples were sent to the National Institute of Virology, Pune. On March 6, 2020, AIIMS, Raipur started performing the RT-PCR tests and served as a mentor to medical institutions in the state to set up molecular virology laboratories. The number of laboratories conducting RT-PCR tests increased from zero to 14 (nine governmental and five private) by June 2021. By September 2020, the state established TrueNAT testing laboratories in all 28 districts. By the end of March 2020, Chhattisgarh tested 71 samples, whereas, by July 2021, the state had conducted more than 11.4 million tests. The daily testing average increased from 8,880 per day in August 2020 to 48,290 in April 2021. The monthly TPR was the highest in April 2021 (26%), followed by September 2020 (15%) and October 2020 (10.5%) (Appendices).

**Figure 2:**
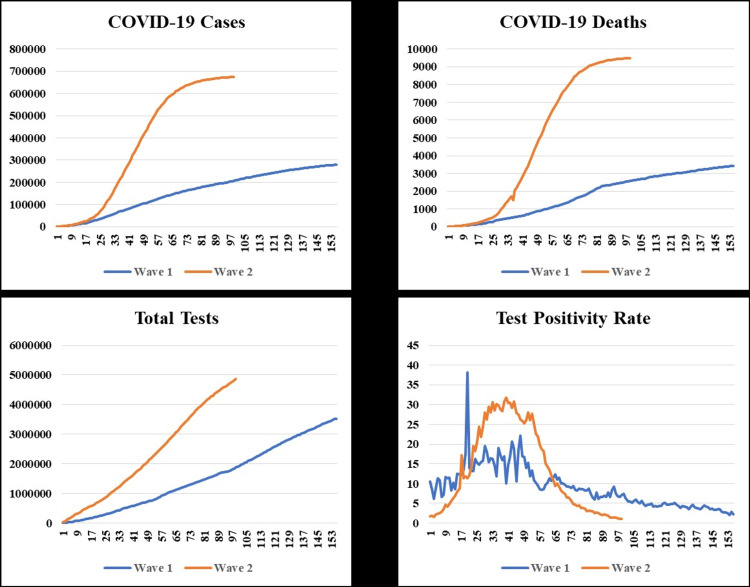
COVID-19 cases, deaths, total tests, and test positivity rates in Chhattisgarh comparing the two waves. The X-axis represents the number of days elapsed during the pandemic wave. Wave 1 lasted from August 15, 2020, to January 17, 2021. Wave 2 lasted from March 13, 2021, to June 19, 2021.

Table 1 and Figure 2 present a comparative analysis of the characteristics of the state’s first and second waves of the pandemic. In the second wave, the number of cases (2.4-fold) and deaths (2.7-fold) increased more than double, and the duration of the second wave decreased by 1.5 times than the first wave. The tests conducted were 1.4 times the previous wave. There was a significant increase in cases per million (p < 0.001), deaths per million (p < 0.001), and TPR (p < 0.001) in the second wave. However, 13 tests were conducted per case during the first wave, which was reduced to seven tests per case during the second wave. The CFR increased from 1.2% in the first wave to 1.4% in the second wave (p < 0.001; odds ratio (OR) = 1.13 (1.1-1.18)). CFR of both males and females significantly increased in the second wave. The reported cases and deaths among males were significantly higher in both waves. The analysis of CFRs in different age groups showed increased mortality in all ages except the young (≤20 years) and the old (>60 years). While 33 out of 100 cases were hospitalized during the first wave, there was a significant decline (p < 0.001), with only 20 out of 100 being hospitalized in the second wave. Although the proportion of patients hospitalized was less in the second wave, in absolute numbers, hospitalized patients were 1.4 times more during the second wave.

**Table 1:** Comparison of characteristics between the first and second waves of COVID-19 in Chhattisgarh. # Percentage of total deaths in age group/ Total number of cases in the age group. * In 31 cases age not known P-values of <0.001 are statistically significant. COVID-19: coronavirus disease 2019

	First wave	Second wave	Odds ratio	P-value
Duration	August 15, 2020 to January 17, 2021 (156 days)	March 13, 2021 to June 19, 2021 (99 days)		
Total number of cases	278,942	674,012		
Total number of deaths	3,437	9,441		
Total tests done	3,517,637	4,874,005		
Test positivity rate	7.90%	13.80%	1.86 (1.85–1.87)	<0.001
Case fatality ratio	1.20%	1.40%	1.13 (1.09–1.18)	<0.001
Percentage hospitalized	33% (n = 91,902)	20% (n = 134,802)		
Cases per million	9,462	22,864	2.4 (2.3–2.47)	<0.001
Deaths per million	118	321	2.7 (2.2–3.3)	<0.001
Test per case	12.6	7.2		
Test per million	119,326	165,337		<0.0001
Gender				
Male (%)	175,706 (63%)	390,826 (58%)	2.5 (2.56–2.59)	<0.001
Female (%)	103,188 (37%)	283,012 (42%)	1.5 (1.49–1.51)	<0.001
Transgender	48	173		
Age (years)*	Cases (% among total cases)	Positive (% among total cases)	OR	
0–10	9,763 (3.5%)	25,612 (3.8%)	2.3 (2.2–2.3)	<0.001
11–20	28,731 (10.3%)	76837 (11.4%)	1.8 (1.8–1.88)	<0.001
21–30	69,178,(24.8%)	163,111 (24.2%)	1.9 (1.9–1.9)	<0.001
31–40	62,762 (22.5%)	153,675 (22.8%)	1.8 (1.8–1.9)	<0.001
41–50	46,304 (16.6%)	117,278 (17.4%)	1.8 (1.8–1.86)	<0.001
51–60	36,820 (13.2%)	81,555 (12.1%)	1.7 (1.7–1.7)	<0.001
61–70	18,131 (6.5%)	39,767 (5.9%)	1.8 (1.7–1.8)	<0.001
71–80	5,858 (2.1%)	12,806 (1.9%)	1.92 (1.8–1.9)	<0.001
>80	1,395 (0.5%)	3,370 (0.5%)	2.05 (1.9–2.2)	<0.001
Age-wise case fatality ratio#				
0–10	0.10%	0.10%	4.7 (2.2–9.9)	<0.001
11–20	0.20%	0.10%	2.2 (1.4–3.3)	<0.001
21–30	0.10%	0.30%	10.5 (8.4–13.1)	<0.001
31–40	0.40%	0.90%	14.5 (12.6–16.7)	<0.001
41–50	1.20%	1.80%	9.5 (8.6–10.4)	<0.001
51–60	2.50%	2.90%	5.7 (5.2–6.2)	<0.001
61–70	4.90%	4.70%	4.5 (4.2–4.9)	<0.001
71–80	8.80%	7.10%	3.9 (3.5–4.4)	<0.001
>80	12.40%	9.50%	4.6 (3.7–5.6)	<0.001

In both waves, the rapid antigen tests (RAT) were widely used, and the proportion of RAT among the COVID-19 tests remained consistent between 60% and 70% of the total tests (Table 2). The government laboratories bore the burden of RT-PCR testing through the pandemic. There was a marginal increase in private laboratory RT-PCR testing in the second wave.

**Table 2:** Proportion of RT-PCR and RAT tests out of the total COVID-19 tests conducted in Chhattisgarh. RT-PCR: reverse transcriptase-polymerase chain reaction; RAT: rapid antigen test; COVID-19: coronavirus disease 2019

	First wave		Second wave	
	Government labs	Private labs	Government labs	Private labs
RT-PCR	32%	1%	26%	3%
RAT	67.3%	0.2%	70.4%	0.3%

Spread in rural areas

**Figure 3:**
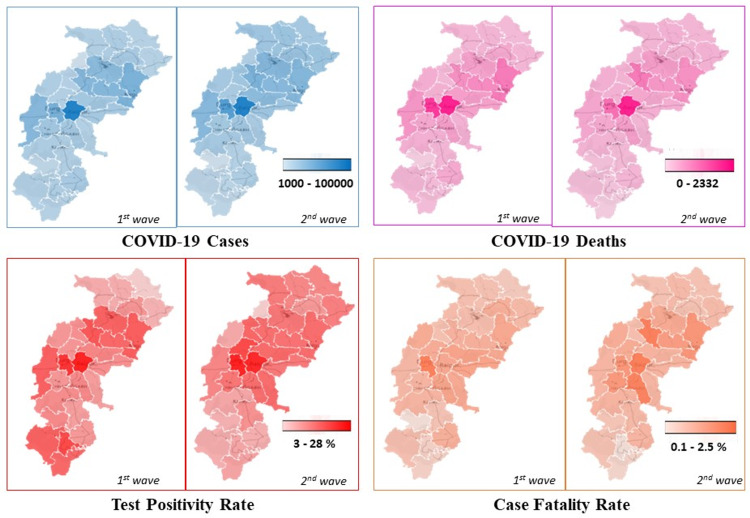
COVID-19 metrics in the first and second waves of the pandemic: distribution in various districts of Chhattisgarh. COVID-19: coronavirus disease 2019

In the first wave, the significant districts with a cumulative TPR of more than 10 were prominent urban localities of Chhattisgarh (Raipur, Durg, Bilaspur, Raigarh, and Rajnandgaon). Dantewada also exhibited high positivity mainly due to positivity in the battalion camps of the defense forces who are stationed in the Naxal-affected areas of the Bastar region. In the second wave, 19 districts had a TPR of more than 10. These included urban districts of Durg, Raipur, Raigarh, Bilaspur, Rajnandgaon, and other predominantly rural districts (Balodabazar, Dhamtari, Gariyaband, Mahasamund, Bemetara, Balod, Mungeli, Janjgir Champa, Korba, Korea, Surajpur, Jashpur, Balrampur, and Kondagaon). The entire five administrative divisions of the state were affected in the second wave (Figure 3, Appendices).

Mucormycosis

Another challenge that the second wave of COVID-19 brought along was the rising incidence of mucormycosis. Although no official figures about the incidence of mucormycosis in the first wave of the pandemic exist, India carried over 70% of the global burden of mucormycosis in COVID-19 patients from December 2019 to April 2021 [7]. Around 15,000 cases of fungal infection have been reported in India by the end of May 2021 [8]. The state of Chhattisgarh reported 276 cases and 17 deaths due to mucormycosis till June 10, 2021 [9]; no case was reported during the first wave.

## Discussion

Sweeping the world in multiple waves, the COVID-19 pandemic has disrupted healthcare, financial, and government systems globally. Despite the deployment of vaccines as early as one year into the pandemic, the mainstay of control continues to be non-pharmaceutical public health intervention measures, enforcing physical distancing, and limiting public activity. However, public fatigue and economic disruptions serve as major challenges in implementing these measures as the pandemic prolongs. A dynamic modeling study that evaluated the impact of multiple parameters such as initial reproduction number, relaxation rate, intervention rate, and fatigue rate concluded that a strong second wave is linked to the absence of resolute response when the disease activity starts increasing after a plummeting period [10]. An assessment of government responses across multiple pandemic waves in 186 countries showed that government responses and stringency of implementation of non-pharmaceutical interventions have a statistically significant association with reduction in COVID-19 incidence and death, enduring across several waves [11].

Most parts of the world such as Africa [12], Europe [13], Japan [14], and Korea [15] have experienced a larger subsequent wave of the pandemic. However, a drop in the CFR and a shift to younger age groups have been commonly reported [16-18]. Western Europe witnessed a strong first wave associated with high R0, affecting older age groups and resulting in high CFRs, while the second wave has affected all ages with much lesser mortality. In Eastern Europe, the first wave was weak but the second was strong and with considerably higher CFR than in the countries further West [10].

Much of the difference in the epidemiological and clinical characteristics of subsequent waves of the pandemic has been related to the variants of the virus that have arisen in different geographic locations. The delta variant (B.1.617.2) of the virus which was first identified in December 2020 has been implicated to be the central cause of the catastrophic COVID-19 surge in India. With increased transmissibility to the tune of twice the wild type, the variant has quickly spread to large parts of the world, literally ushering in a new phase in the pandemic [19]. A decreased effectiveness after one dose of vaccine (ChAdOx1 nCoV-19, Covishield, and BNT162b2, Pfizer-BioNTech) has been noted with the delta variant compared to the alpha variant [20]. Fortunately, the reduction in vaccine effectiveness after two doses has been modest [20]. A study based on model-inference methods to reconstruct COVID-19 pandemic dynamics in India concluded that the delta variant was able to escape wild-type immunity almost half the time and was 60% more infectious than the latter [21]. Among the other reasons cited for the disastrous second wave in India are the large population, incoherent containment strategies, poor air quality index, violation of COVID-19-appropriate behavior, and widespread vaccine hesitancy [3,22].

This article describes and compares the two waves of the COVID-19 pandemic in Chhattisgarh, a central Indian state. Both waves were triggered by increased social activity and relaxed norms, as has happened everywhere in the world. The first wave lasted over five months and had a much lower peak than the second wave which was sharp and explosive with almost thrice the number of cases and deaths. The odds of dying during the second wave were 14 times higher than in the first wave. The age group of 21-50 had higher mortality odds. Notably, our findings have refuted the speculations of an increased CFR in the young population. Additionally, in the elderly population, a slight but significant decrease in mortality was seen which could be attributable to vaccination in that age group that was initiated during the onset of the second wave. The mortality rate in males, especially in the age group of >50 was higher. This is in concordance with the findings of a meta-analysis by Biswas et al. [23].

The second wave exhibited a markedly greater TPR, shooting as high as 33%, compared to the first wave’s peak of 20%. However, the test per case (12.6 and 7.2 for the first and second waves, respectively) for the two waves has remained much lower than the recommendation by the World Health Organization of 20-30. The reliance on RAT testing has also been high throughout the pandemic, as has been the case with most other Indian states. Throughout the first and second waves, the proportion of RT-PCR tests among total tests in the private laboratories increased marginally due to the dearth of accredited laboratories in Chhattisgarh.

The percentage of cases hospitalized in the second wave dropped sharply. MoHFW issued a series of guidelines and revisions on home care or home isolation which were widely acceptable and preferred modes of treatment for the general community. This may be one of the reasons for the relatively less proportion of hospitalizations during the second wave. With the huge numbers testing positive and healthcare setups getting overwhelmed, hospital beds were available only to the sick and desaturating patients during the peak of the second wave.

The second wave was also marked by much more extensive involvement of the state’s rural districts. A commendable job was done by the government and state administration by quickly imposing lockdowns in districts with a TPR of >8. Public health measures were promptly put in place and vaccination was strengthened. As of August 13, 2021, Chhattisgarh has administered over 1.28 crore doses of the vaccine against SARS-CoV-2, with 86% of the population above 45 years of age [24] and 28% in the age group 18-44 having received the first dose [25]. The state is testing about 40,000 samples daily on an average, and the current TPR is less than 0.5% [26]. Continued awareness and epidemiological monitoring are crucial.

The delta variant of the virus that has been ascribed to be the central cause for the COVID-19 surge in India [3] accounted for three-fourths of the sequenced samples in the second pandemic wave in Chhattisgarh. Out of the 940 SARS-CoV-2-positive samples sent for whole-genome sequencing from the state from the onset of the second wave (March 13, 2021, to June 02, 2021), 736 (74.5%) were delta variants. The number of samples sequenced from the state has been limited, but the available data point to the fact that the second wave was largely attributable to the delta variant [25].

The fourth nationwide COVID-19 serosurvey conducted between June 14 and July 06, 2021, indicated that about two-thirds of India’s population had antibodies against SARS-CoV-2 after the massive second wave compared to 24% from December 2020 to January 2021 [27]. This rise in seroprevalence could be attributed to either natural infection or vaccination. Notably, seroprevalence among unvaccinated adults increased from 24.3% in December 2020-January 2021 to 62.3% in June-July 2021, indicating that it was a natural infection during the second pandemic wave in the months of March-June 2021 that was largely responsible for the increase in seroprevalence. Another striking finding of this study was the comparable seroprevalence in rural and urban areas, which attests to the fact the second wave had hit the rural areas equally hard.

Vaccination in India began in January 2021 and was expanded in a phase-wise manner. After the frontline workers and the elderly, the third phase of vaccination targeting the 18-44-year age group was launched on May 1, 2021, which coincided with the peak of the second wave in most Indian states. Along with the high number of cases and deaths, the nation was facing a shortage of vaccines. The state of Chhattisgarh, like many others, faced similar glitches in the vaccination program, especially during the months when the second wave of the pandemic had hit due to supply logistics, public hesitancy, and certain government policies [28,29]. Hence, there was no immediate benefit of the vaccination program in reducing the second peak. By the end of June 2021, Chhattisgarh could administer 2,531,466 first doses and 69,839 second doses of the vaccine to the 18-44-year age group [30]. The vaccination drive could progress in its full swing only after the second wave subsided just like the rest of the country.

Study limitations

This study has some limitations. As there is no standard definition of a wave, the duration of the first and second waves was based on assumptions of a rolling average of more than 500 cases. Thereafter, asymptomatic cases are less likely to get tested. This may have impacted the overall profile such as age, gender, and geographical distribution of the cases. Widespread use of RAT leading to false negativity and duplication of data due inclusion of repeat tests are some of the additional limitations. Future studies with case-specific information such as clinical features, comorbidities, treatment modalities, and outcomes would be helpful. Analysis of the effect of vaccination has not been conducted in this study as the COVID-19 vaccination commenced in the second wave in phases, initially for health workers and frontline workers, followed by community members with comorbidity and age more than 45, and, lastly, the general community. The study may overall be limited in terms of completeness of data availability, especially pertaining to the delta variant and mucormycosis.

## Conclusions

Despite the limitations, this comprehensive analysis of the COVID-19 epidemiology provides an insight into the nature of the pandemic in Chhattisgarh (located in the central part of India), evaluating key differences between the two waves. The second wave of COVID-19 was more precarious than the first, although mortality among the young did not increase. In the second wave, the number of cases (2.4-fold) and deaths (2.7-fold) increased more than double, and the duration of the second wave decreased by 1.5 times than the first wave. The tests conducted were 1.4 times the previous wave. There was a significant increase in cases per million (p < 0.001), deaths per million (p < 0.001), and TPR (p < 0.001) in the second wave. Regular surveillance, monitoring, and analysis of epidemiological data may help formulate informed policies and intervention strategies.

## Appendices

**Table 3:** Age-specific incidence and death rates for COVID-19 in Chhattisgarh. $ Last year of age group has been added to the next age group for ease of calculation. For example, for the age group of 0-10 years, 0-9 is available and age 10 is added to the next age group, i.e., 11-20 years). COVID-19: coronavirus disease 2019

Age group	COVID-19 cases in the first wave	COVID-19 cases in the second wave	COVID-19 incidence in the first wave	COVID-19 incidence in the second wave	COVID-19 deaths in the first wave	COVID-19 deaths in the second wave	COVID-19-specific death rate in the first wave	COVID-19-specific death rate in the first wave	Population as per Census 2011$
0–10	9,763	25,612	0.2%	0.5%	11	20	0.00%	0.00%	5,299,823
11–20	28,731	76,837	0.5%	1.4%	49	41	0.00%	0.00%	5,483,855
21–30	69,178	16,3111	1.6%	3.7%	99	442	0.00%	0.01%	4,446,797
31–40	62,762	15,3675	1.7%	4.3%	230	1,373	0.01%	0.04%	3,594,472
41–50	46,304	11,7278	1.6%	4.0%	548	2,070	0.02%	0.07%	2,910,927
51–60	36,820	81,555	2.1%	4.6%	915	2,373	0.05%	0.13%	1,782,300
61–70	18,131	39,767	1.4%	3.1%	897	1,859	0.07%	0.15%	1,277,552
71–80	5,858	12,806	1.1%	2.4%	515	912	0.10%	0.17%	539,578
81 and more	1,395	3,370	0.7%	1.8%	173	320	0.09%	0.17%	186,779

**Table 4:** Monthly COVID-19 metrics. COVID-19: coronavirus disease 2019; CFR: case fatality ratio

Month	Cases	Recovered	Deaths	Tests	Case/Million	Test/Million	Recovery rate	Cumulative CFR	Monthly CFR	Cumulative SPR	Monthly SPR
March 2020	9	2	0	787	0	27	22%	0.0%	0.0%	1.1%	1.1%
April 2020	31	34	0	16,754	1	568	110%	0.0%	0.0%	0.2%	0.2%
May 2020	458	78	4	51,611	16	1c,751	17%	0.2%	0.9%	0.7%	0.9%
June 2020	2,413	2,136	14	91,498	82	3,104	89%	0.5%	0.6%	1.8%	2.6%
July 2020	6,224	4,070	51	155,477	211	5,274	65%	0.6%	0.8%	2.9%	4.0%
August 2020	22,311	10,669	377	266,413	757	9,037	48%	0.9%	1.7%	5.4%	8.4%
September 2020	82,099	64,729	1,178	524,072	2,785	17,778	79%	0.8%	1.4%	10.3%	15.7%
October 2020	73,668	81,361	755	702,834	2,499	23,842	110%	1.1%	1.0%	10.3%	10.5%
November 2020	50,052	51,747	542	749,433	1,698	25,422	103%	1.2%	1.1%	9.3%	6.7%
December 2020	42,226	49,943	503	951,111	1,432	32,264	118%	1.2%	1.2%	8.0%	4.4%
January 2021	19,753	32,570	286	705,597	670	23,935	165%	1.2%	1.4%	7.2%	2.8%
February 2021	7,193	8,612	132	591,969	244	20,081	120%	1.2%	1.8%	6.5%	1.2%
March 2021	36,627	13,114	414	945,530	1,242	32,075	36%	1.2%	1.1%	6.1%	3.9%
April 2021	379,513	282,096	4,411	1,448,692	12,874	49,143	74%	1.1%	1.2%	7.9%	26.2%
May 2021	242,763	32,1513	4,467	1,918,743	8,235	65,088	132%	1.1%	1.8%	107.9%	12.7%
June 2021	23,017	52,403	391	1,224,994	781	41,555	228%	1.1%	1.7%	207.9%	1.9%

**Table 5:** Supplementary data on cases regarding age and gender.

	First wave positive cases	First wave negative cases	Second wave positive cases	Second wave negative cases	Odds ratio	Positivity in the first wave	Positivity in the second wave	Proportion in the first wave	Proportion in the second wave
Age (years)									
0–10	9,763	151,220	25,612	170,321	2.3 (2.27–2.38)	6%	13%	4%	4%
11–20	28,731	378,351	76,837	545,117	2.3 (2.2–2.3)	7%	12%	10%	11%
21–30	69,178	988,668	163,111	1,193,188	1.8 (1.8–1.88)	7%	12%	25%	24%
31–40	62,762	666,287	153,675	863,327	1.9 (1.9–1.9)	9%	15%	23%	23%
41–50	46,304	422,807	117,278	581,056	1.8 (1.8–1.9)	10%	17%	17%	17%
51–60	36,820	280,428	81,555	356,188	1.8 (1.8–1.86)	12%	19%	13%	12%
61–70	18,131	141,317	39,767	172,032	1.7 (1.7–1.7)	11%	19%	6%	6%
71–80	5,858	38,183	12,806	43,280	1.8 (1.7–1.8)	13%	23%	2%	2%
>80	1,395	9,722	3,370	11,446	1.92 (1.8–1.9)	13%	23%	1%	0%
Gender									
Male	175,706	1,739,801	390,826	1,498,163	2.5 (2.5–2.6)	9%	21%	63%	58%
Female	103,188	1,335,200	283,012	2,433,646	1.5 (1.49–1.51)	7%	10%	37%	42%
